# A rationale and framework for sharing mindfulness in trauma-dense communities in South Africa

**DOI:** 10.4102/safp.v67i1.6114

**Published:** 2025-06-09

**Authors:** Simon Whitesman

**Affiliations:** 1 Institute for Mindfulness South Africa, Cape Town, South Africa; 2 Division of Family Medicine and Primary Care, Faculty of Medicine and Health Sciences, Stellenbosch University, Stellenbosch, South Africa

**Keywords:** mindfulness, mindfulness-based interventions, trauma-sensitive mindfulness, continuous traumatic stress, mindfulness-based pedagogy

## Abstract

Mindfulness has its historical roots in the teachings of the Buddha. The core of these teachings addresses the causes and relief of human suffering. It is a way of being with experience, through awareness of the present moment, in service of compassion and wisdom. Its universal applicability lies in the fact that it is an innate human capacity, which can be developed through practice. Mindfulness has moved into broader contemporary society through the emergence of mindfulness-based interventions (MBIs) over the last 50 years and an associated robust research base. Mindfulness-based pedagogy has been largely restricted to first-world countries although a 2-year training at Stellenbosch University has been an exception to this. Research based on the experience of graduates of this programme has resulted in a new, emergent curriculum that is more context- and trauma-sensitive, to better meet the challenges of the local setting, and to make learning how to share mindfulness with others more accessible, relevant and Afro-centric.

## Introduction

Mindfulness and its derivative vehicles for delivery, mindfulness-based interventions (MBIs), are described in peer-reviewed and popular literature as an effective way of dealing with many of the common ailments of our time: stress, trauma, depression, anxiety and functional physical disorders^1^ as a result of a growing body of scientific evidence.^2^ Mindfulness has also found utility beyond healthcare, including education, politics, business, sports and even the military.^3^

Effectively sharing mindfulness with others is contingent on training teachers and facilitators in a way that is grounded in experiential learning and supplemented by a theoretical understanding of both the science and wisdom-based roots of this contemplative practice. The additional element that has become increasingly relevant in mindfulness-based pedagogy is infusing such training with context sensitivity^4,5^ in a way that retains fidelity to the practice and is congruent with the context.

This has particular relevance within South (and Southern) Africa, given the historical legacy of Apartheid and the pervasive and ongoing impact of trauma on the lives of many in this country. As such, training people to share mindfulness in this environment needs to consider the complex dynamics of the country (and region), while honouring both the universality of mindfulness and the unique attributions of practicing on African soil.

## What is mindfulness?

The term mindfulness first appeared in the English language in 1881 as a translation of the Buddhist term *sati* (Pali) or *smrti* (Sanskrit),^6^ which conveys the sense of ‘remembering’ to pay attention in the present, and also recalling a deeper, inherently aware or awakened sense of self.^6^ Mindfulness emerges from first-person phenomenological experience, extending from the personal to interpersonal and systemic domains.^7^ That is, as awareness develops and refines, it expands to incorporate embodied present experience in a relational and social field. Historically rooted in pre-scientific Buddhist culture, mindfulness was intrinsically linked to introspective awareness aimed at addressing the causes, and relief of human suffering.^7^ In modern contexts, mindfulness remains as relevant as ever in addressing the many and varied expressions of human distress. One of the compelling dimensions of contemporary research in this field – which confirms ancient wisdom teachings – is the degree to which mindlessness is a foundation for this suffering, a state characterised by absent-mindedness or thought wandering.^8^ The recognition of mindlessness enables a ‘waking up’, which Kabat-Zinn^9^ describes as an ‘orthogonal rotation in consciousness’. This realisation disrupts habitual thinking and behavioural patterns, fostering presence of mind, a state of being where thoughts are perceived as transient phenomena within a larger awareness. The re-establishment of present-moment awareness invites and allows for response flexibility, rather than patterned reactivity, based on present reality, rather than imagined past and/or future virtual realities.

Central to mindfulness is its emphasis on here-ness and now-ness, a present-centred awareness often described as a felt sense of being.^10^ Practitioners are encouraged to directly explore this spacious quality of presence, confirming its validity through personal experience.

### The evolution of contemporary mindfulness

Mindfulness has transitioned significantly from its Buddhist origins into what is now termed contemporary mindfulness. This transformation has been influenced by two key developments:

- Western practitioners immersing themselves in Buddhist practices in Asia and subsequently adapting and sharing these teachings for secular audiences in the West.^3^
- The introduction of Mindfulness-Based Stress Reduction (MBSR) into a tertiary teaching hospital in Massachusetts in 1979, which significantly broadened mindfulness’s accessibility to people who would otherwise have been unlikely to have discovered or practiced.^11^

Contemporary mindfulness often faces criticism from Buddhist scholars who argue that modern interpretations may dilute or decontextualise its ethical and wisdom components.^12^ However, Kabat-Zinn^13^ defends this evolution as a recontextualisation, ensuring mindfulness remains relevant and practical in secular contexts.

Efforts to define mindfulness in contemporary terms aim to strike a balance between traditional Buddhist wisdom and operational functionality. Bishop et al.^14^ proposed a widely accepted two-component model of mindfulness, comprising:

- Self-regulation of attention, fostering sustained focus on present experiences.
- Attitudinal qualities of openness, curiosity and acceptance towards these experiences.

### Mindfulness as a universal human capacity

Mindfulness transcends cultural or religious specificity, emerging as a universal human capability. It is not an activity but a state or quality of being in relationship with experiences. Mindfulness evolves, and is refined, through cultivation, progressing from self-awareness to self-regulation and ultimately to self-transcendence, where personal concerns expand to include prosocial intentions and the wish for the well-being of others.^15^ This universality underscores mindfulness’s accessibility to all individuals willing to engage with it intentionally.

## Practical applications and impact

The practical utility of mindfulness lies in its potential to alleviate suffering. Mindfulness-Based Stress Reduction (MBSR), developed by Jon Kabat-Zinn, and first offered at a tertiary teaching hospital in Worcester, Massachusetts in 1979, exemplifies this by providing a structured framework for teaching mindfulness in a medical setting. Originally developed for patients with chronic illnesses, MBSR has demonstrated significant benefits,^11^ inspiring adaptations like Mindfulness-Based Cognitive Therapy (MBCT) for preventing relapse in depression.^16,17^ Such adaptations highlight mindfulness’s adaptability across diverse contexts, including health,^18^ education,^19^ workplace,^20^ and politics.^21^

Mindfulness-based interventions are rooted in Kabat-Zinn’s operational definition, emphasising purposeful, present moment awareness imbued with non-judgement in service of understanding, wisdom and compassion. The core elements of formal mindfulness practices (such as sitting meditation and body scanning), integrating moments of mindfulness into daily living, the use of enquiry to investigate direct experience (with the support of a teacher) and context-specific didactic input (such as the cognitive model of depressive relapse and the dynamics of chronic stress) are what characterise MBIs irrespective of the population or setting. This foundation ensures mindfulness remains practical while preserving its transformative potential.

Mindfulness, while universally recognised and increasingly applied, encompasses a rich and multifaceted tradition. Its evolution from Buddhist teachings to secular applications illustrates its adaptability and relevance. Mindfulness is more than a cognitive or therapeutic technique; it is a profound way of being that fosters self-awareness, ethical engagement and a compassionate relationship with lived experience. Harnessing mindfulness’s transformative potential requires maintaining its integrity while ensuring accessibility across diverse societal domains.

## Trauma-sensitive adaptations in South Africa

In South Africa, systemic trauma – shaped by historical, socio-political and economic inequities – creates a unique context for MBIs. Effective mindfulness teaching requires sensitivity to diverse sociocultural contexts and the potential trauma histories of participants, particularly in environments characterised by continuous stress or adversity.^22^ This trauma-sensitivity must guide the pedagogy and delivery of mindfulness, placing compassion, embodiment and relational safety at its core. A safe relationship is one which is emotionally attuned, non-reactive, with clear boundaries and a non-judgemental attitude. Kabat-Zinn’s view that MBIs are a ‘re-contextualisation’ of wisdom traditions for modern society supports the notion of further adapting these practices for local contexts.^13^

Mindfulness practitioners, teachers and facilitators in South Africa often operate in trauma-dense communities where continuous traumatic stress challenges traditional mindfulness delivery models.^23^ To address these realities, a pedagogical framework that emphasises trauma-awareness and the integration of compassionate, embodied presence into mindfulness training is essential to allow wider access and uptake of this universal practice, and as much and wherever possible, mitigate the risk of re-traumatisation.

## Evolving frameworks for teaching and training

Globally, access to MBIs has predominantly benefitted white, middle-class populations, neglecting marginalised communities.^4^ South Africa’s socio-economic disparities necessitate adaptations to ensure mindfulness practices reach under-resourced populations. Training curricula must address systemic inequities by empowering local facilitators and incorporating culturally relevant practices.

The current South African mindfulness training model, which has been largely based on curricula developed in first-world settings, has not sufficiently addressed these disparities. Research exploring the experience of graduates of a 2-year training at Stellenbosch University who taught mindfulness in trauma-dense environments has formed the basis of a revision of the curriculum that closer aligns with the realities of past and continuous trauma in South Africa.^5^ A key modification of the pedagogical framework includes practices and learning experiences that prioritise creating emotionally attuned and responsive relationships, nervous system regulation and the development of self-compassion. This revised approach seeks to mitigate the dissociative tendencies often associated with trauma and to enhance participants’ ability to re-engage with their somatic experiences.^24^ In addition, the creation of a 3-month training model which is delivered online rather than in-person, reduces the costs and, as such, increases the potential access to the training from more diverse sectors of society.

## Balancing benefits and risks in trauma-dense environments

Teaching mindfulness in environments of continuous traumatic stress demands a careful balance between potential benefits and risks. Trauma disrupts awareness and attachment bonds, leading to protective (adaptive) dissociation, in which there is a disruption in the awareness and integration of thoughts, feelings and embodied experience.^25^ This adaptative process, over time, creates maladaptive symptoms many of which become embedded in, or expressed through the body, such that the ‘body keeps the score’.^26^ Learning and practicing mindfulness involves developing somatic awareness, given that ‘mindfulness of the body’ is the foundation of the original wisdom teachings on mindfulness.^27^ The convergence of these dimensions may reintroduce distressing sensations into consciousness. Facilitators must skilfully support participants in navigating these experiences within a safe, regulated environment.

Trauma-sensitive practices involve creating a secure holding space through ‘security priming’ – cultivating feelings of safety and connectedness.^28^ This approach regulates the nervous system, activating the soothing system associated with relational calm and resilience.^29,30^ Compassion, a cornerstone of mindfulness practice, emerges naturally in such an environment, generating psychological and physiological benefits.^31^

## An evolving pedagogy

The dynamic adaptation of mindfulness pedagogy in South Africa reflects a broader need for innovation in low- and middle-income countries (LMICs). It requires not only curricular fluidity, which retains integrity to the approach, but also engagement with a wider range of stakeholders that reflect the contextual demographics, both professional and non-professional, especially within the health, social development and educational sectors. Continuous learning and participatory engagement are essential for developing context-sensitive approaches that retain fidelity to mindfulness’s foundational principles.^32^ The emerging curriculum prioritises relational safety, compassionate presence and systemic equity, creating a model for delivering mindfulness in under-resourced and trauma-affected communities.

Informed by the experiences of South African mindfulness practitioners and global insights, this revised pedagogy underscores the transformative potential of mindfulness when grounded in cultural relevance and trauma-awareness. As Santorelli^33^ notes, the evolution of mindfulness training is an ongoing process, driven by the need to remain responsive and attuned to emergent challenges and opportunities.

## Curriculum design for greater inclusivity

The revised training curriculum introduces and foregrounds relational and regulatory practices, within a sensitive holding environment, facilitated by experienced teacher-trainers. Trauma-sensitive mindfulness practices are complemented by techniques to promote nervous system regulation. This learning environment is conducive to practicing, experimenting and applying mindfulness in its various forms, before sharing them with others.

Layers of support are provided through teacher-led whole group sessions, mentor-led small groups and through leaning partner dyads. These spaces support the facilitator-in-training to explore their personal mindfulness practice, reflect on theoretical resources, to explore contextual issues and to experiment with guiding trauma-sensitive practices in feedback with experienced teachers and peers. The graduates then have a range of brief, trauma-sensitive practices that are familiar and internalised to share with the communities in which they work.

A core teaching intention is for the teacher-trainers to embody and teach compassionate presence, encouraging participants to internalise these qualities, which they, in turn, will embody for those with whom they share these practices.

Language, cultural context and inclusivity are central to the revised approach. Training sessions integrate trauma-sensitive language and culturally resonant practices and resources to foster a distinctly African flavour. These adaptations ensure mindfulness remains accessible and relevant to the diverse realities of South African communities.

## Conclusion

Mindfulness is a universally accessible, innate human capacity. Various lines of research have shown its broad efficacy and impact in health and well-being. As such, the practices lend themselves to integration in our society, given the high level of need to provide as many people as possible with internal resources that can help to address the various levels and expressions of suffering that beset our country. An innovative, accessible, context- and trauma-sensitive curriculum has been created to deliver training to those who wish to learn how to share mindfulness with others in a context-congruent way.
